# Acetyl‐11‐keto‐beta‐boswellic acid modulates macrophage polarization and Schwann cell migration to accelerate spinal cord injury repair in rats

**DOI:** 10.1111/cns.14642

**Published:** 2024-03-02

**Authors:** Yao Wang, Zongliang Xiong, Yuncong Qiao, Qiyuan Zhang, Guanghu Zhou, Chong Zhou, Xianglin Ma, Xiaowen Jiang, Wenhui Yu

**Affiliations:** ^1^ Department of Veterinary Medicine Northeast Agricultural University Harbin Heilongjiang China; ^2^ School of Life Sciences Northeast Agricultural University Harbin Heilongjiang China; ^3^ Key Laboratory of the Provincial Education, Department of Heilongjiang for Common Animal Disease Prevention and Treatment Northeast Agricultural University Harbin Heilongjiang China; ^4^ Institute of Chinese Veterinary Medicine Northeast Agricultural University Harbin Heilongjiang China

**Keywords:** AKBA, inflammatory markers, macrophage polarization, Nrf2/HO‐1 signaling pathway, SCI, spinal cord injury

## Abstract

**Background:**

Inhibiting secondary inflammatory damage caused by glial cells and creating a stable microenvironment is one of the main strategies to investigate drugs for the treatment of spinal cord injury. Acetyl‐11‐keto‐beta‐boswellic acid (AKBA) is the active component of the natural drug boswellia, which has anti‐inflammatory and antioxidant effects and offers a possible therapeutic option for spinal cord injury.

**Methods:**

In this study, a spinal cord injury model was established by crushing spinal cord, respectively, to detect the M1 macrophage inflammatory markers: iNOS, TNF‐α, IL‐1β, and the M2 macrophage markers CD206, ARG‐1, IL‐10, and the detection of antioxidant enzymes and MDA. In vitro, macrophages were cultured to verify the main mechanism of the macrophage switch from Nrf2/HO‐1 to M2 type by flow cytometry, immunofluorescence, and other techniques. Macrophage and Schwann cell co‐culture validated the migration mechanism of Schwann cells promoted by AKBA.

**Results:**

AKBA significantly enhanced the antioxidant enzyme activities of CAT, GSH‐Px, T‐AOC, and SOD, reduced MDA content, and reduced oxidative damage caused by spinal cord injury via the Nrf2/HO‐1 signaling pathway; AKBA mediates Nrf2/HO‐1/IL‐10, converts macrophages from M1 to M2 type, reduces inflammation, and promotes Schwann cell migration, thereby accelerating the repair of spinal cord injury in rats.

**Conclusions:**

Our work demonstrates that AKBA can attenuate oxidative stress as well as the secondary inflammatory injury caused by macrophages after SCI, promote Schwann cell migration to the injury site, and thus accelerate the repair of the injured spinal cord.

## Graphical abstract

Acetyl‐11‐keto‐beta‐boswellic acid (AKBA) can protect against spinal cord injury by regulating the redox system Nrf2/HO‐1 signaling pathway, reducing the ROS generation, and promoting the migration of macrophages to M2 polarization and Schwann cells.

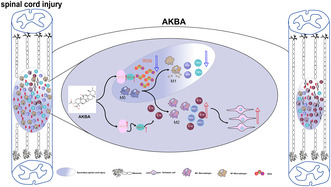

## 1 INTRODUCTION

Central nerve injury is followed not only by neuronal cell death but also by local microglia activation and macrophage invasion, mediating immune responses and neuroinflammation^1^ that cause secondary spinal cord injury. The activation of macrophages and microglia results in two types of cells, M1 and M2.^2^ M1 macrophages secrete large amounts of pro‐inflammatory factors such as inducible nitric oxide synthase (iNOS),^3^ which promote the development and persistence of inflammation, while M2 macrophages secrete Interleukin 10 (IL‐10) and other inflammation‐inhibiting factors.^4^ This facilitates the repair of damage. It has been shown that increased expression of Tumor Necrosis Factor‐α (TNF‐α) contributes to the transformation of macrophages into M1,^5^, ^6^ which secretes the toxic substance iNOS and produces inflammation. In contrast, Interleukin‐1 beta (IL‐1β) is the main pro‐inflammatory factor in the spinal cord, generating a harmful microenvironment in injured tissues and expanding the extent of the injury.^7^ Macrophage mannose receptor 1 (CD206) and Arginase‐1 (ARG‐1) are M2 macrophage markers, and IL‐10 is an anti‐inflammatory substance secreted by M2, which can resist the inflammatory attack on cells.^8^ M1 to M2 polarization of macrophages after spinal cord injury is beneficial to reduce spinal cord injury.^9^ And polarized macrophages contribute to the migration of Schwann cells.^10^ In the central nervous system (CNS), Schwann cells have a role in promoting the axonal regeneration and myelin repair.^11^ Therefore, the migration of Schwann cells contributes to the repair of spinal cord injury.^12^, ^13^

Reactive oxygen species (ROS) are signaling molecules essential for the progression of the immune response,^14^ and nuclear factor E2‐related factor 2 (Nrf2) is a key nuclear factor in the cellular defense mechanism against oxidative stress, activating downstream antioxidant enzymes such as heme oxygenase 1 (HO‐1) to protect cells from oxidative damage and inflammation generated by ROS stimulation.^15^ It has been shown that Nrf2 plays a key role in the immune system, limiting the inflammatory response by inhibiting ROS and the pro‐inflammatory cytokines IL‐1β, and IL‐6.^16^ It has also been shown that Nrf2/HO‐1 regulates the M2‐like polarization of macrophages, thereby promoting neural regeneration and functional recovery from injury.^17^

AKBA with molecular formula C_32_H_48_O_5_ is a pentacyclic triterpenoid that the 11 keto groups can effectively inhibit 5‐lipoxygenase.^18^ It is demonstrated that AKBA additionally activates cellular 15‐LOX‐1 via an allosteric site accomplishing robust Specialized pro‐resolving mediators (SPM) formation in innate immune cells, particularly in M2 macrophages, further promoting the regression of inflammation.^19^ It is shown that AKBA can upregulate Nrf2, downregulate NF‐κB, reduce oxidative stress and inflammation.^20^, ^21^ It has been shown that AKBA can promote sciatic nerve injury and repair.^22^, ^23^, ^24^ It has also been shown to reduce experimental autoimmune encephalomyelitis through Nrf2.^25^ We hypothesized a view: “AKBA can regulate the polarization of macrophages via Nrf2/HO‐1 and accelerate the repair of spinal cord injury”. In view of this hypothesis, we used the spinal cord crush injury model to explore whether AKBA can reduce the activation of M1 macrophages, increase the activation of M2 macrophages, promote the migration of Schwann cells, reduce microenvironmental inflammation, and protect the spinal cord from secondary injury. Our results provide a basis for traditional medicine frankincense in the treatment of nerve injury.

## 2 MATERIALS AND METHODS

### 2.1 Fabrication of animal models and drug delivery methods

Forty SD female rats (180–200 g) (Liaoning Changsheng Biotechnology Co., Ltd, China; license No. SCXK (Liao) 2020‐0001) were randomly divided into four 4 groups: sham operation group (S), sham operation administration group (S + A), model group (M), and model administration group (M + A). The spinal cord injury model was performed by aneurysm clip extrusion. The specific operation Kjell as described^26^: Rats were anesthetized with Zoletil 50 (30 mg/kg; Virbac, Nice, France) at the posterior end of the T6‐T7 spine, perpendicular to the muscle and fascia cut with the intervertebral disc space. The spine and posterior lamina of T6‐T7 were cut just enough to expose the spine and posterior lamina of T6‐T7, and some tissue was removed to expose a segment of the spinal cord and perform a laminectomy. The spinal cord injury model was created using the aneurysm compression method.^27^ In the sham‐operated group, only the spinal cord was exposed without spinal cord compression. After the operation, the rats were transferred to the pre‐prepared cages and the status of the rats was observed daily, and successful modeling was considered when the rats dragged their hind limbs. The sham‐operated and model‐administered groups were given AKBA (Shanghai yuanye Bio‐Technology Co. Ltd, Shanghai, China) 20 mg/kg^28^ by gavage every day. The other groups were gavaged with the same amount of solvent. A quantity of 80 mg of AKBA was weighed and dissolved in 1 mL of dimethyl sulfoxide (DMSO) (80 mg/mL) as the mother liquor. Then configured in this ratio (5% DMSO + 30% polyethylene glycol 300 + 65% distilled water) as 4 mg/mL of gavage solution, the dose administered to rats was 0.5 mL/100 g. All animal experiments were approved by the animal ethics committee of Northeast Agricultural University under approval number neauec2022 05 08.

### 2.2 Behavior

Spinal cord injuries were tested for science using the Basso, Beattie & Bresnahan (BBB) locomotor rating scale.^29^ To reduce the error of the results, we used a double‐blind method in the experiment, that is, two people observed and recorded independently. Then take the average value.

### 2.3 Hematoxylin–eosin staining (H.E.) staining

On day 7, the rats were anesthetized via intraperitoneal injection of Zoletil 50 (30 mg/kg) and sacrificed by cervical dislocation. The spinal cord was surgically exposed and the spinal cord at the site of injury was isolated. The spinal cord (*n* = 3/group) was fixed with 4% paraformaldehyde, and after 1 day of fixation, H.E. staining was performed, in brief, wax dipping, embedding, making paraffin sections, and observation under a light microscope.

### 2.4 Immunofluorescence

The above paraffin sections (*n* = 3/group) were placed in a repair box filled with ethylenediaminetetraacetic acid (EDTA) disodium salt (pH 8.0) antigen repair solution and subjected to antigen repair in a microwave oven. After natural cooling, the slides were placed in phosphate‐buffered saline (PBS) (pH 7.4) and washed 3 times. The sections added bovine serum albumin (BSA) dropwise and closed for 30 min. Then the sections were prepared according to Nrf2, s‐100, iNOS, CD206 rabbit antidilution (1:200). And covered with CY3/AF488 goat anti‐rabbit diluent (1:300) dropwise and incubated for 50 min at room temperature. Nucleus was restained with 4′,6‐diamidino‐2‐phenylindole (DAPI) and photographed with an ortho‐fluorescence microscope (Nikon Eclipse C1). The results were analyzed using image J.

### 2.5 Cell culture

Macrophages were cultured using the RAW264.7 cell line (CL‐0190, Procell Life Science & Technology, Wuhan, China) and Rma‐bm (R1920, Yaji Biological, Shanghai, China), starting from generation 4. Rat Schwann cells were cultured using the RSC96 cell line (CL‐0199, Procell Life Science & Technology, Wuhan, China) for passaging and tested starting from the 4th generation. The RAW264.7 cell and RSC cells medium consisted of 10% FBS (Clark Bioscience, Virginia, USA) and DMEM (Meilun, Dalian, China) and 1% triple antibodies (Meilun, Dalian, China) containing penicillin, streptomycin, and amphotericin. The Ram‐bm cell culture medium is a special culture medium for Ram‐bm, purchased from Yaji biological.

### 2.6 Establishment of LPS induced macrophage polarization model and the determination of optimal AKBA administration concentration

Firstly, the cells were divided into four groups: blank group (C), blank administration group (A), lipopolysaccharide (LPS) group (L), and LPS + AKBA group (L + A). Secondly, after passage of RAW264.7 cells, they were cultured in a cell incubator. Following Dong's method, the M1 type model of macrophages was induced with 1 μg/mL LPS.^10^ To determine the optimal drug concentration for AKBA treatment of RAW264.7 cells, the CCK8 method was used to determine the cell viability of LPS (1 μg/mL) and AKBA treated cells at different concentrations. Dissolve AKBA in DMSO to prepare AKBA mother solution (8 mg/mL), and then use Dulbecco's modified Eagle's medium (DMEM) gradient dilution to prepare AKBA working solution. RAW264.7 cells were subcultured until the 4th generation. Cell count, as 2 × 10^5^ cells/mL, at a rate of 100 μL cells per well add to the 96 well plate and set a blank group without cells. Incubate in a cell incubator for 12 h, remove the 96 well plates, and add AKBA working solutions of 0, 0.3125, 0.625, 1.25, 2.5, 5, 10, and 20 μg/mL, with 6 replicates in each group for 12 h. After 12 h, add Cell Counting Kit‐8 (CCK8) reagent. After 1 h, measure the absorbance at 560 nm using an enzyme‐linked immunosorbent assay. The optimal dosage of AKBA acting on macrophages was screened through CCK8.

### 2.7 Typing test of macrophages

The RAW264.7 macrophage M1 model was created with 1 μg/mL LPS. It has been shown that AKBA can promote the expression of Nrf2, so the group with the addition of Nrf2 inhibitor (ML385) (5 μm)^30^ (MedChemExpress, Monmouth Junction, NJ, USA) was designed. Cells were divided into the control group, control + AKBA group, control + ML385 group, LPS group, LPS + AKBA group, and LPS + AKBA + ML385 group. After passing the cells and waiting for 6 h to adhere to the wall, the serum‐free medium was replaced with starvation culture for 6 h, followed by LPS, AKBA and ML385 pretreatment for 12 h, and cells were collected for WB after 12 h of action (3 replicates in each group).

The Rma‐bm macrophage M1 model was created with 1 μg/mL LPS. It has been shown that AKBA can promote the expression of Nrf2, so the group with the addition of Nrf2 inhibitor (ML385) (5 μm) (MedChemExpress, Monmouth Junction, NJ, USA) was designed. Cells were divided into the control group, control + ML385 group, control + AKBA group, control + AKBA + ML385 group, LPS group, LPS + AKBA group, LPS + ML385 group, and LPS + AKBA + ML385 group. The optimal concentration of the AKBA group is 2.5 μm. After passing the cells and followed by LPS, AKBA and ML385 pretreatment for 12 h, and cells were collected for WB after 12 h of action (3 replicates in each group).

### 2.8 Cellular immunofluorescence assay

After cell passaging, plates were spread and added to 24‐well plates at 500 μL per well and were treated according to 2.6 (3 replicates in each group). Then fixed overnight with 4% paraformaldehyde. The next day, three washes with PBS for 5 min each, followed by the addition of 2.5% Triton X‐100 for 20 min, then three washes with PBS, then 5% goat serum for 30 min at room temperature. Then added primary antibody of iNOS or CD206 was in 24‐well plates, 4°C overnight incubation, and secondary antibody and DAPI incubation the next day. Finally, photos were taken by fluorescence microscopy.

### 2.9 ELISA kits and ROS kit

The levels of oxidative stress in spinal cord tissue were detected using an ELISA kit consisting of CAT, GSH‐PX, T‐AOC, SOD, and MDA. Using TNF‐α, IL‐1β, iNOS ELISA kit tested the levels of inflammatory factors in the serum. The specific experimental operations are strictly carried out according to the reagent kit. Cell cultures were collected for kit testing. TNF‐α, IL‐1β, and iNOS kits were performed according to kit instructions. According to the instruction manual, use the ROS kit (Nanjing jiangcheng Bioengineering Insitiute, China) for ROS fluorescence detection, take pictures with a fluorescence microscope, and image‐J for analysis.

### 2.10 Flow cytometry analysis

After the pretreatment as described in method 2.6, the cells were cultured, and the cells were collected in the tube with a cell scraper. To check the expression of F4/80 and CD206 in the cells, fix in 4% paraformaldehyde for 30 min, then incubated with F4/80 containing PAC (Invitrogen, Thermo Fisher, USA) and PE CD206 (Invitrogen, Thermo Fisher, USA) containing PE for 1 h at room temperature. Finally, cells were washed three times with ice‐cold PBS and used in 500 μL PBS for analysis. 10^4^ cells from each sample were analyzed using a BD FACSCanto II cytometer (Becton Dickinson, San Jose, CA).

### 2.11 Cell migration assay

Collect the macrophage culture medium in method 2.6 in advance and store it at −80°C. The matrigel matrix gel (Corning, Bedford, MA) was put from −20°C to liquid state overnight in 4°C degree refrigerator. The Matrigel was diluted 1:8 and wrapped on the upper chamber side of the membrane at the bottom of the transwell. The RSC96 cells were cultured to the 4th generation and then made into 10^5^ cells/mL cell suspension. A volume of 200 μL of the cell suspension was added to the transwell. A volume of 500 μL of macrophage culture medium (3 replicates in each group) was added to the lower chamber of the 24‐well. The cells were cultured for 24 h and then stained. The chambers were fixed with poly methanol for 30 min, air‐dried properly, stained with 0.1% crystal violet for 30 min, and washed 3 times with PBS. Photographs were taken under a microscope.

### 2.12 Western blot

Weigh 1 g each of day 7 spinal cord (*n* = 3/group), add 1 mL of lysis solution containing 0.1% PMSF and 0.1% phosphorylated protease inhibitor Radio Immunoprecipitation Assay (RIPA) and grind with tissue grinder, lyse on ice for 30 min after grinding, centrifuge at 12,000 r/min for 15 min, take 300 μL of supernatant. The gels were configured by spotting 5 μL of the uniform sample, running the upper gel at 80 V and the lower gel at 120 V. Then cut the gel according to the protein size, transfer the membrane, close it, and incubate the primary antibody, see the primary antibody information Table 1. After 12 h incubation of the primary antibody, the membrane is washed 3 times with TBST, the rabbit antibody is incubated, and the membrane is washed again for exposure. The Tanon 5200 gel imaging system is used for imaging, and image‐J is used for gray‐scale analysis.

**TABLE 1:** Information of primary antibodies.

Antibodies	Concentration	Species	Catalog no.	RRID no.	Supplier
IL‐1β	1:500	Rabbit	WLH3903	AB_2894981	Wanleibio (Shenyang, China)
TNF‐α	1:500	Rabbit	WL01581	AB_2894992	Wanleibio (Shenyang, China)
β‐Actin	1:1000	Rabbit	bs‐0061R	AB_10855480	Bioss (Beijing, China)
ARG‐1	1:500	Rabbit	WL02825	AB_2732850	Wanleibio (Shenyang, China)
IL‐10	1:1000	Rabbit	WL03088	AB_764545	Wanleibio (Shenyang, China)
NRF2	1:1000	Rabbit	bs‐1074R	AB_10855421	Bioss (Beijing, China)
HO‐1	1:1000	Rabbit	bs‐2075R	AB_10857174	Bioss (Beijing, China)
CD206	1:200	Rabbit	orb4941	AB_10922121	Biorbyt (Cambridge, UK)
INOS	1:200	Rabbit	bs‐0162R	AB_10855051	Bioss (Bei‐jing, China)
S‐100	1:200	Rabbit	bs‐1248R	AB_563998	Bioss (Beijing, China)

### 2.13 Real‐time fluorescent quantitative polymerase chain reaction (RT‐qPCR)

Total RNA was extracted according to chi's method,^31^ and the RNA concentration was measured by nanodrop. After the OD value was between 1.9 and 2.0, HiScript III RT SuperMix for RT‐qPCR (+gDNA wiper) kit (Vazyme Biotech Co, Ltd) for reverse transcription, after reverse transcription, real‐time fluorescent quantitative PCR was performed. The list of primers is shown in Table 2. Use BioEasy Master Mix (Probe, High ROX) (Hangzhou Bioer Technology, China) dyes and primers, template premixed, then add 10 μL per well into a 96‐well PCR plate, and perform detection on a Roche 480 machine according to the program provided on the dye. Results Data processing was performed according to 2^−ΔΔt^,^32^ and the ratio of the target gene to glyceraldehyde‐3‐phosphate dehydrogenase (GAPDH) was obtained.

**TABLE 2:** Primer sequence list.

Genes	Primer sequence
*ARG‐1*	(F) 5′‐AGAGGCTCGCAGGGAAGA‐3′
	(R) 3′‐GCTGTCATTGGGGACATCCA‐5′
*IL‐1β*	(F) 5′‐TTG AGT CTG CAC AGT TCC CC‐3′
	(R) 3′‐GTC CTG GGG AAG GCA TTA GG‐5′
*TNF‐α*	(F) 5′‐ACT GAA CTT CGG GGT GAT CG‐3′
	(R) 3′‐GCT TGG TGG TTT GCT ACG AC‐5′
*Nrf2*	(F) 5′‐GTCCCAGCAGGACATGGATTT‐3′
	(R) 3′‐AGCGACTGAAATGTAGGTGAAG‐5′
*HO‐1*	(F) 5′‐GTCCCAGCAGGACATGGATTT‐3′
	(F) 5′‐GTCCCAGCAGGACATGGATTT‐3′
*IL‐10*	(F) 5′‐AATTGAACCACCCGGCATCT‐3′
	(R) 3′‐TTTCCAAGGAGTTGCTCCCG‐5′
*GAPDH*	(F) 5′‐GGA GAT TAC TGC CCT GGC TC‐3′
	(R) 3′‐GAT GGT GAT GGG TTT CCC GT‐5′

### 2.14 Statistical analyss

Statistical analysis was performed using GraphPad Prism 8.0 (GraphPad Software, San Diego, CA, USA). The data is first confirmed to have a normal distribution using the SPSS.22 description method. After all data conforms to a normal distribution, the data is analyzed and statistically analyzed using one‐way ANOVA. Results are represented as mean ± standard (*n* = 3/group) deviation, and significance was analyzed using one‐way‐ANOVA, with statistically significant differences at *p* < 0.05 indicated with *, *p* < 0.01 indicated with **, *p* < 0.001 indicated with ***, *p* < 0.0001 indicated with ****.

## 3 RESULTS

### 3.1 AKBA can promote the functional recovery after spinal cord injury

**FIGURE 1:**
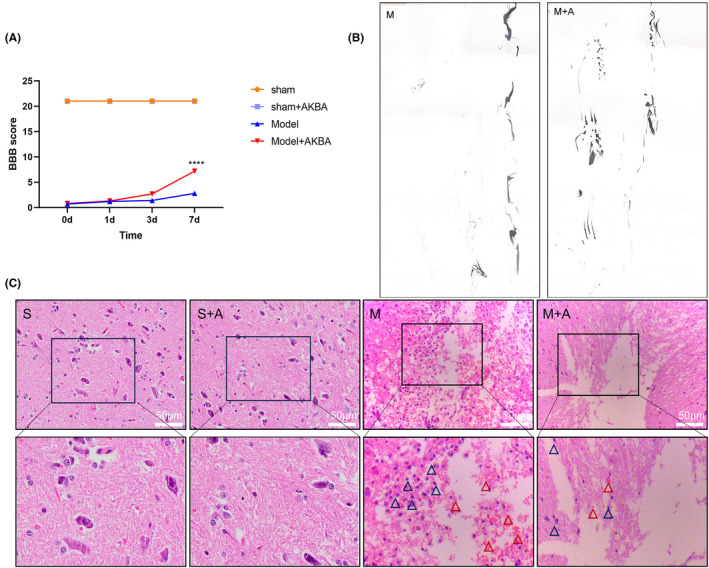
AKBA can promote the reduction of inflammation and the promotion of functional recovery after spinal cord injury. (A) 7‐day detection score chart of BBB. (B) Rat footprint detection chart of Model group and Model + AKBA group. (C) HE histopathological detection chart. The blue triangle represents the inflammatory cells, and the red triangle represents the bleeding points. “S” represents the Sham group, “S + A” represents the Sham + AKBA group, “M” represents the spinal cord injury model group, and “M + A” represents the model + AKBA group. ****p < 0.0001.

On day 3 day, the BBB scale showed no significant difference between the model group and the AKBA administration group, but on day 7 a significant difference appeared between the model group and the AKBA administration group, and the AKBA administration group showed better functional recovery than the model group (Figure 1A). Figure 1B shows that the model group dragged, while the rats tended to walk after AKBA administration, but still dragged. The HE staining in the figure showed that the inflammatory cell infiltration was severe in the model group, while the inflammatory infiltration was significantly reduced after the administration of AKBA (Figure 1C).

### 3.2 AKBA mediates inflammation reduction by iNOS with CD206

**FIGURE 2:**
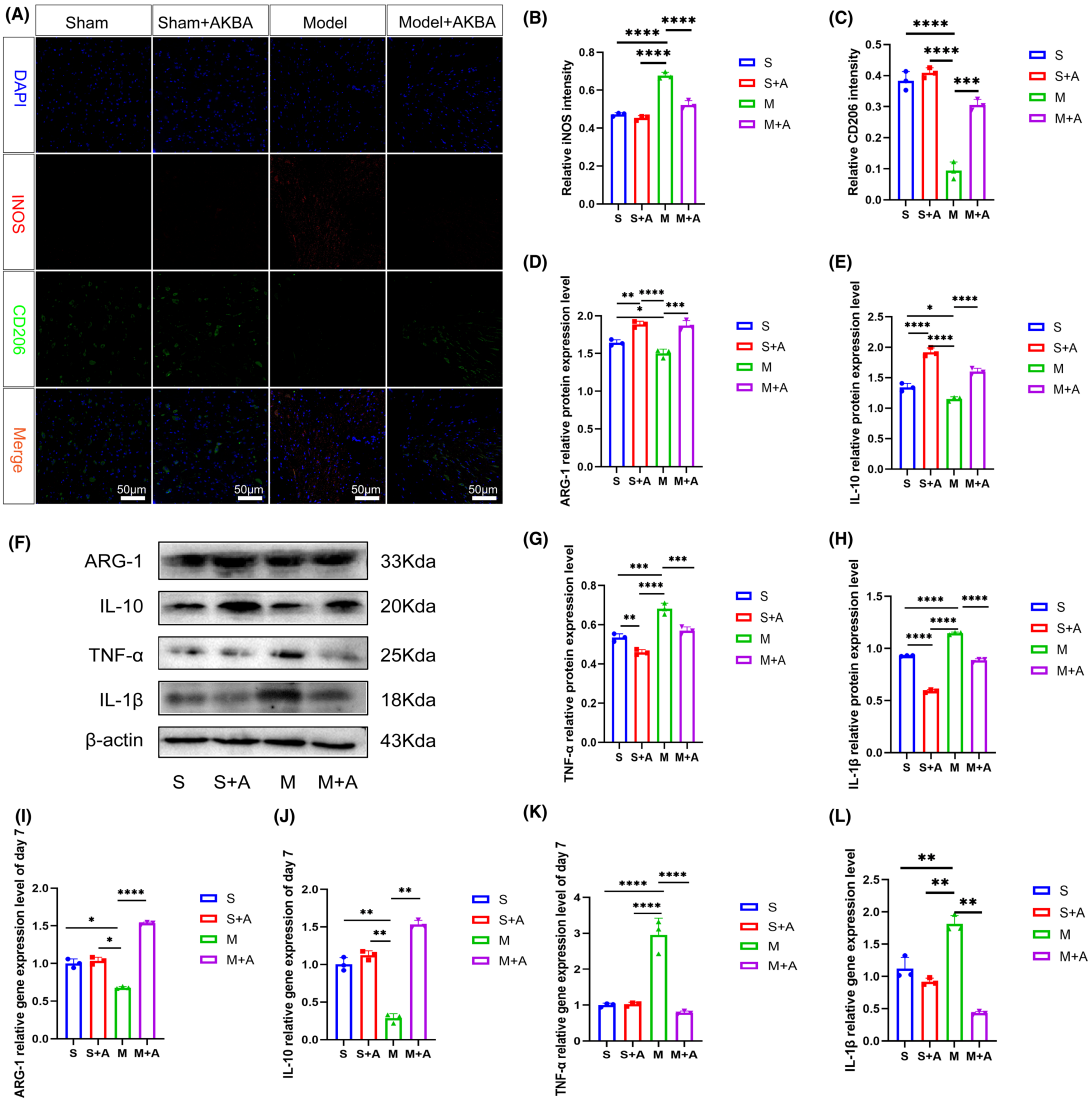
AKBA reduces inflammation by reducing the expression of iNOS. (A) Fluorescent staining pictures of iNOS and CD206. Red is iNOS, green is CD206. The white scale is 20 μm. (B) iNOS fluorescence semi‐quantitative analysis. (C) Fluorescence semiquantitative analysis of CD206. (D) Protein quantitative analysis of ARG‐1. (E) Protein quantitative analysis of IL‐10. (F) Protein expression map of pro‐inflammatory and anti‐inflammatory factors. (G) Protein quantification of TNF‐α. (H) Protein quantitative analysis of IL‐1β. (I–L) Gene expression levels of ARG‐1, IL‐10, TNF‐α, and IL‐1β. *p < 0.05, **p < 0.01, ***p < 0.001, ****p < 0.0001.

INOS is a cytotoxic substance released by microglia and macrophage activation, and its accumulation causes secondary damage to other cells. On day 7, as shown in Figure 2A,B, the expression of iNOS was significantly upregulated in the model group compared with the sham‐operated group, while after the addition of AKBA, the expression of iNOS was significantly downregulated compared with the model group. The expression of inflammatory factors TNF‐α and IL‐1β was significantly upregulated in the model group compared with the sham‐operated group, while the expression of TNF‐α and IL‐1β was significantly downregulated after the addition of AKBA. It indicates that AKBA can reduce inflammation (Figure 2F–H). TNF‐α and IL‐1β gene levels were also significantly down‐regulated after the addition of AKBA (Figure 2K,L).

While CD206 is a marker of M2‐like glial cells and macrophages, it is an anti‐inflammatory phenotype. On day 7, the fluorescence of CD206 was significantly decreased in the model group compared with the sham‐operated group, while the fluorescence intensity was significantly upregulated after the addition of AKBA compared with the model group, as shown in Figure 2A,C. The expression of anti‐inflammatory factors ARG‐1 and IL‐10 was significantly downregulated in the model group compared with the sham‐operated group, while it was significantly upregulated after the addition of AKBA compared with the model group (Figure 2D–F). The gene expression levels of ARG‐1 and IL‐10 were also significantly up‐regulated after the addition of AKBA (Figure 2I,J).

### 3.3 AKBA can activate the Nrf2 pathway to reduce the oxidative stress after spinal cord injury

**FIGURE 3:**
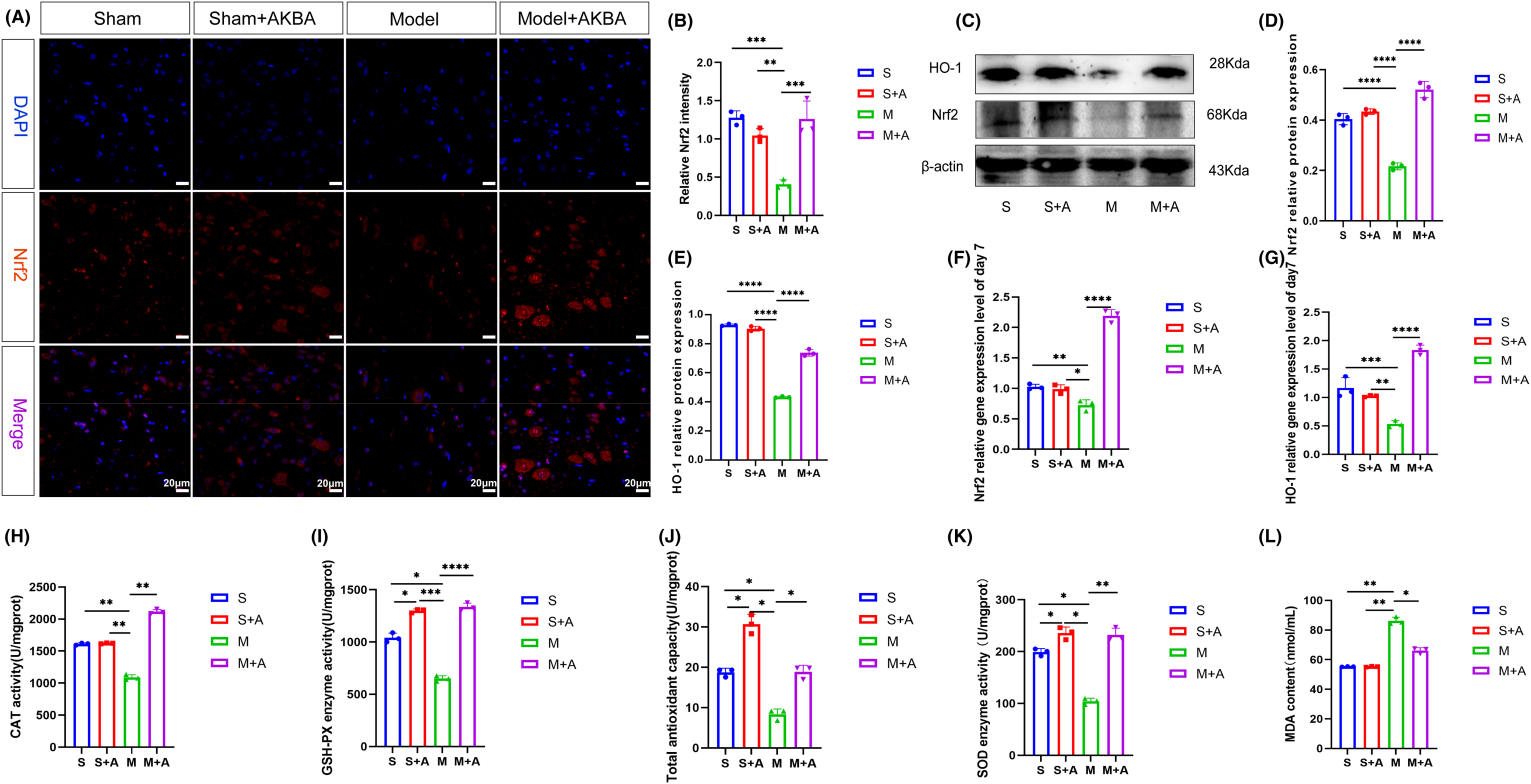
AKBA can activate the expression of Nrf2 pathway to reduce oxidative stress. (A) Pictures of the fluorescent expression of Nrf2 in the spinal cord. The white scale is 20 μm. (B) Quantitative analysis of Nrf2 fluorescence pictures on day 7. (C) Nrf2/HO‐1 protein expression map. (D) Quantitative analysis of Nrf2 protein on day 7. (E) Quantitative analysis of HO‐1 protein on day 7. (F) The mRNA relative expression level of Nrf2 on the 7th day. (G) Relative mRNA expression level of HO‐1 on day 7. (H) CAT content. (I) GSH‐PX content. (J) T‐AOC content. (K) SOD enzyme activity. (L) MDA content. *p < 0.05, **p < 0.01, ***p < 0.001, ****p < 0.0001.

Nrf2 is an important antioxidant factor in vivo, and its activation can translocate to the nucleus to cause the release of antioxidant enzymes and promote the repair of damage. On day 7, as described in Figure 3A,B, the fluorescence expression of Nrf2 was reduced in the model group compared with the sham‐operated group, and the fluorescence expression of Nrf2 was enhanced after the addition of AKBA treatment, and all of them were located in the nucleus. As shown in Figure 3C–G, the AKBA group significantly increased the protein expression level and gene expression level of Nrf2 and HO‐1 compared with the model group. And the results of CAT, GSH‐PX, T‐AOC, and SOD antioxidant enzymes showed that AKBA significantly increased the activity of antioxidant enzymes and reduced the production of MDA (Figure 3H–L), thus protecting the spinal cord and accelerating spinal cord injury repair.

### 3.4 AKBA reduces the expression of LPS‐induced macrophage pro‐inflammatory phenotype

**FIGURE 4:**
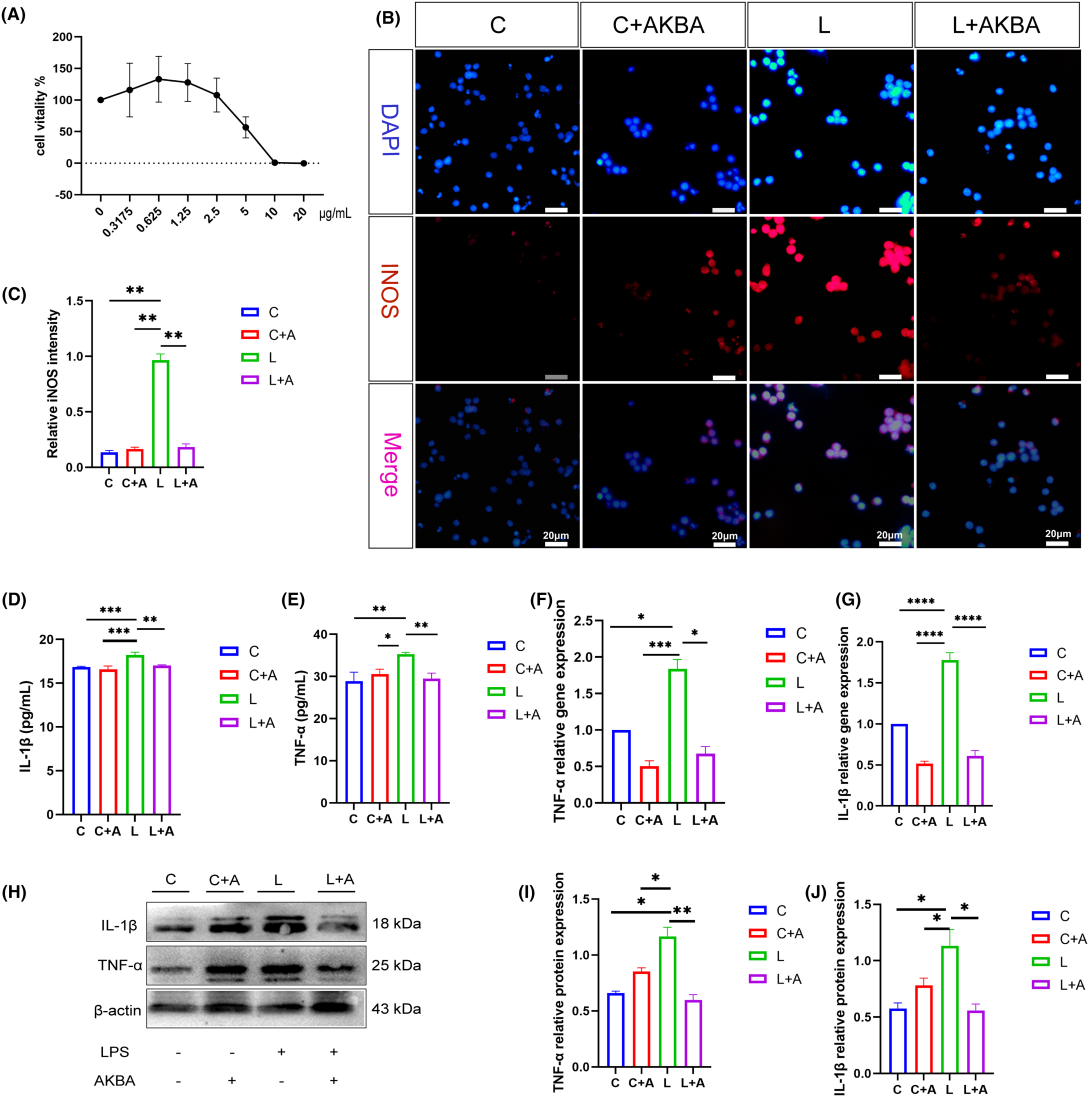
AKBA can reduce the expression of macrophage pro‐inflammatory phenotype induced by LPS. (A) AKBA optimal concentration. (B) The fluorescence expression map of iNOS. The white scale is 20 μm. (C) Fluorescence quantitative analysis diagram of iNOS. (D) The expression level of TNF‐α detected by ELISA. (E) The expression level of IL‐1β detected by ELISA. (F, G) Gene expression levels of TNF‐α, IL‐1β. (H) Protein expression levels of TNF‐α, IL‐1β. (I, J) Protein quantitative analysis of TNF‐α, IL‐1β. *p < 0.05, **p < 0.01, ***p < 0.001, ****p < 0.0001.

To further verify whether AKBA can promote the transformation of macrophages and reduce inflammation. As shown in Figure 4A, we chose 0.625 μg/mL as the optimal concentration for AKBA to act on macrophages. Figure 4B,C, compared with the LPS group, the fluorescence intensity of iNOS was significantly reduced after adding AKBA. As shown in Figure 4D,E, compared with the LPS group, the expressions of TNF‐α and IL‐1β were significantly decreased after AKBA intervention. As shown in Figure 4F–J, compared with the control group, after LPS stimulation, the mRNA expression level and protein expression level of TNF‐α and Il‐1β in the cells were significantly increased, but after adding AKBA significantly lower compared with the LPS group.

### 3.5 AKBA increases the anti‐inflammatory expression and reduces the ROS release in M2 macrophages

**FIGURE 5:**
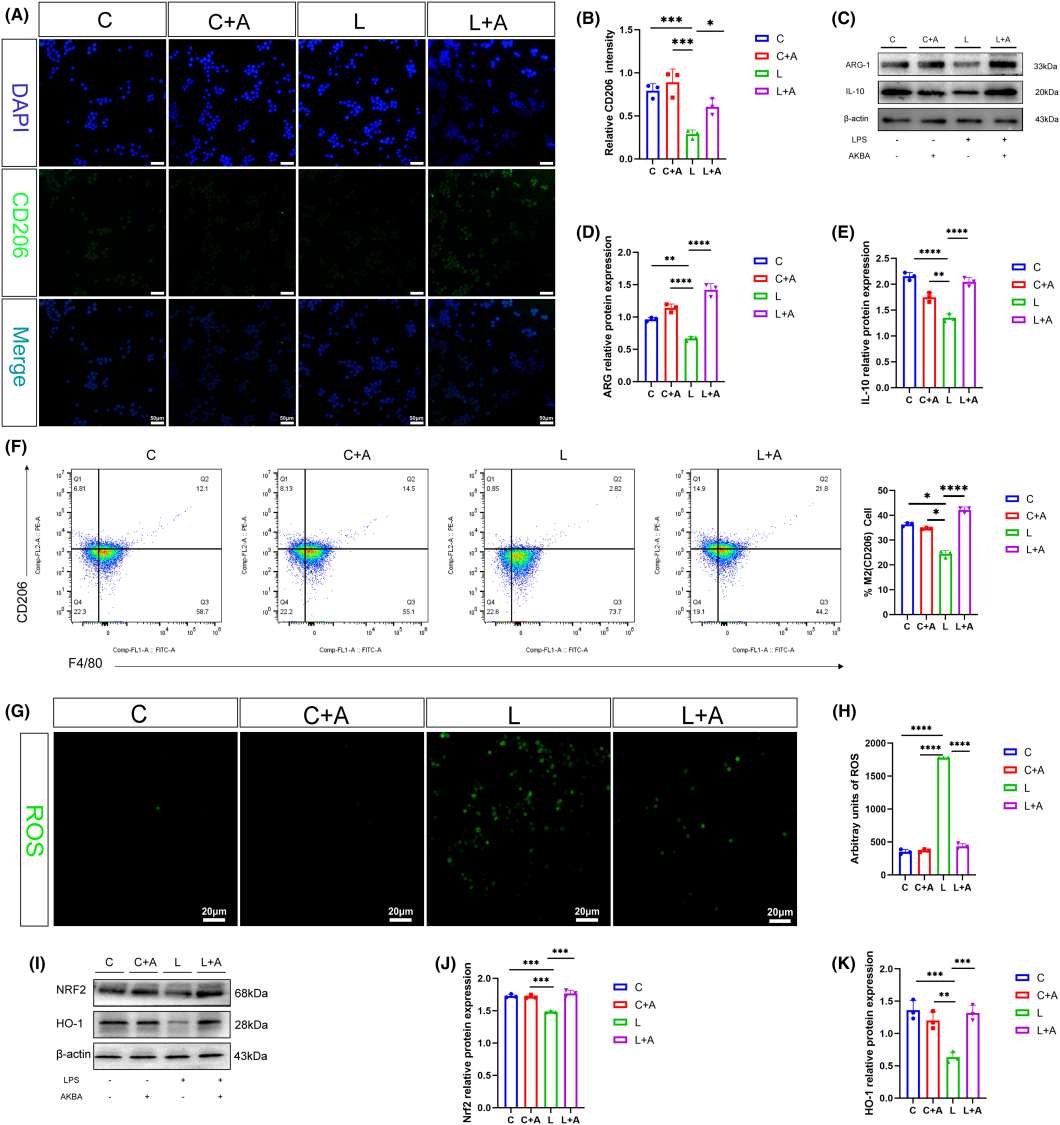
AKBA can increase the anti‐inflammatory expression of macrophages induced by LPS and reduce the release of ROS. (A) Fluorescent expression map of CD206. The white scale is 20 μm. (B) Fluorescence quantitative analysis diagram of CD206. (C) Protein expression map of ARG‐1 and IL‐10. (D) Protein quantitative analysis chart of ARG‐1. (E) Protein quantitative analysis chart of IL‐10. (F) Fluorescence quantitative analysis of ROS. (G) CD206 flow cytometry results. (H) Expression map of ROS. The white scale is 20 μm. (I) Protein expression map of Nrf2 pathway. (J, K) Protein Quantitative Analysis of Nrf2, HO‐1. *p < 0.05, **p < 0.01, ***p < 0.001, ****p < 0.0001.

As shown in Figure 5A,B, after adding AKBA, the fluorescence intensity of CD206 was significantly enhanced. As shown in Figure 5C–E, compared with the control group, after LPS stimulation, the protein expression levels of ARG‐1 and IL‐10 were significantly reduced, but in the LPS + AKBA group, the protein expression levels of ARG‐1 and IL‐10 expression levels were significantly increased. We performed flow cytometric analysis on the cells and found that CD206 was significantly up‐regulated after adding AKBA, which was consistent with the fluorescence results (Figure 5G). We detected the ROS, an important signaling factor. As shown in Figure 5F–H, it was found that after LPS stimulation, the expression of ROS was significantly increased, and after adding AKBA, the expression of ROS could be significantly reduced; To further explore why AKBA promotes macrophage transformation. The expression of Nrf2 and HO‐1 were detected, compared with the blank group, LPS can reduce the expression of Nrf2, HO‐1, and after adding AKBA, compared with the LPS group, the expression of Nrf2, HO‐1 was significantly increased (Figure 5I–K).

### 3.6 The Nrf2 inhibitor ML385 attenuates the pro‐macrophage transformation effect of AKBA

**FIGURE 6:**
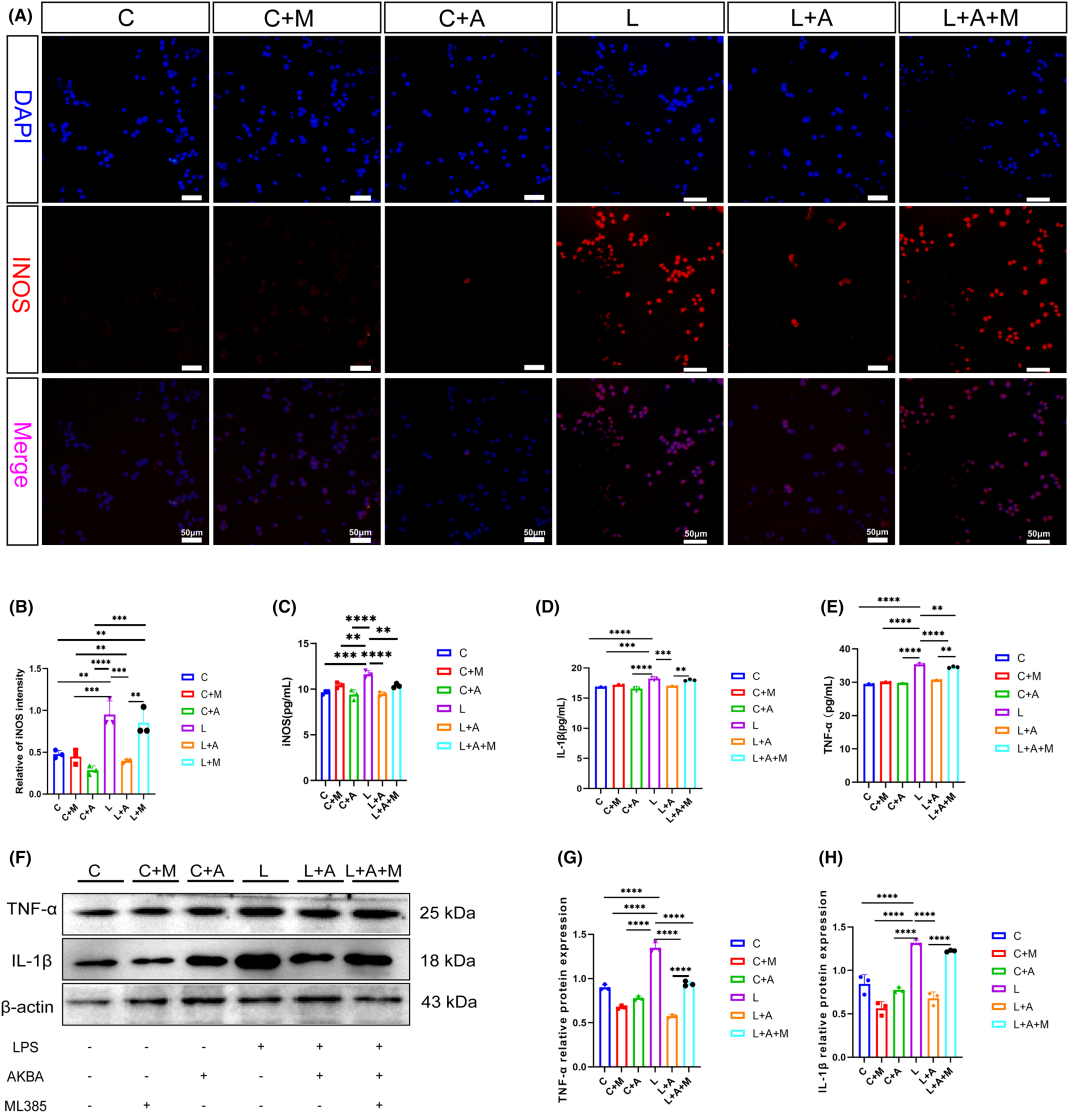
Nrf2 inhibitors attenuate the ability of AKBA to reduce the iNOS expression. (A) The fluorescence expression map of iNOS. The white scale is 20 μm. (B) Fluorescence quantitative analysis diagram of iNOS. (C) The expression level of iNOS detected by ELISA. (D) Detection level of IL‐1β detected by ELISA. (E) The detection level of TNF‐α detected by ELISA. (F) Protein expression map of TNF‐α and IL‐1β. (G, H) Protein quantitative analysis of TNF‐α, IL‐1β. **p < 0.01, ***p < 0.001, ****p < 0.0001.

We used Nrf2 inhibitor (ML385) to determine Nrf2 induced by AKBA whether promotes macrophage transformation. As shown in Figure 6A–C, compared with the LPS + AKBA group, the addition of ML385 again attenuated the ability of AKBA to reduce the expression of iNOS fluorescence, while the same results were observed when ELISA was used to detect the expression of iNOS in the culture supernatant. As shown in Figure 6D–H, the ability of AKBA to reduce iNOS was attenuated after the addition of ML385 inhibitor, and the protein expression of TNF‐α and IL‐1β in culture medium and macrophages was also detected, and the ability of AKBA to attenuate TNF‐α and IL‐1β protein expression was attenuated after the addition of ML385.

### 3.7 Nrf2 inhibitor attenuates AKBA's ability to promote the macrophage transformation and reduce the ROS release

**FIGURE 7:**
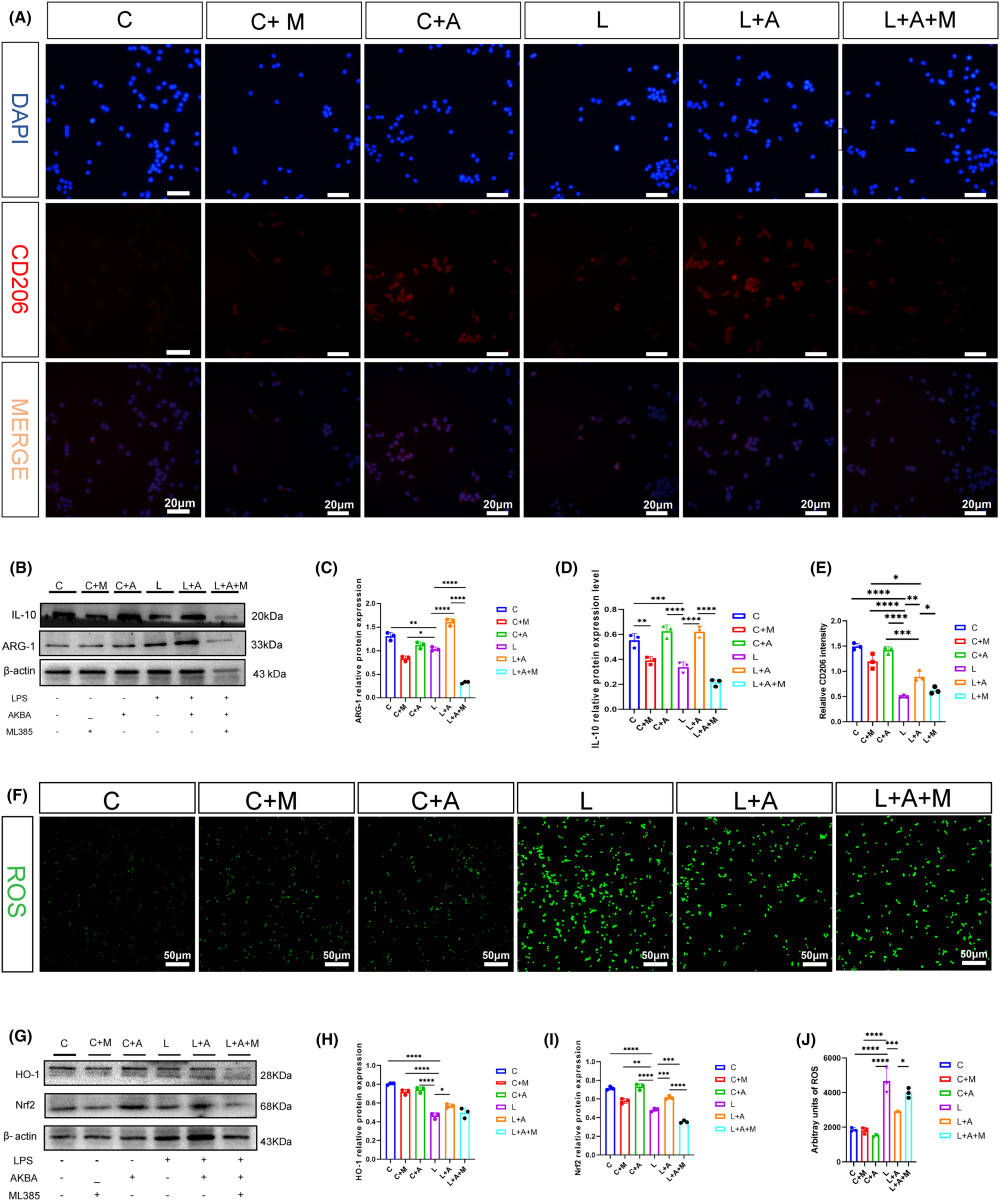
Nrf2 inhibitors attenuate the ability of AKBA to increase the CD206 expression. (A) Fluorescent expression map of CD206. The white scale is 20 μm. (B) Fluorescence quantitative analysis of CD206. (C) Protein expression maps of ARG‐1 and IL‐10. (D, E) Protein quantitative analysis of ARG‐1, IL‐10. (F) Expression map of ROS. The white scale is 50 μm. (G) Protein expression map of Nrf2/HO‐1. (H, I) Protein quantitative analysis of Nrf2 and HO‐1. (J) Quantitative analysis of ROS. *p < 0.05, **p < 0.01, ***p < 0.001, ****p < 0.0001.

As shown in Figure 7A,E, the ability of AKBA to increase CD206 fluorescence expression was attenuated by the addition of ML385 again compared to the LPS + AKBA group. The protein expression of ARG‐1 and IL‐10 in macrophages was also examined, and both obtained that the ability of AKBA to increase the protein expression of ARG‐1 and IL‐10 was diminished after the addition of ML385 (Figure 7B–D).

By detecting the protein expression of Nrf2, HO‐1, we found that the ability of AKBA to increase Nrf2, HO‐1 was diminished after adding the Nrf2 inhibitor (Figure 7G–I). And ROS was detected, and it was found that the ability of AKBA to reduce ROS was inhibited after the addition of inhibitors (Figure 7F,J).

### 3.8 Nrf2 inhibitor attenuates the ability of AKBA to promote the migration of Schwann cells

**FIGURE 8:**
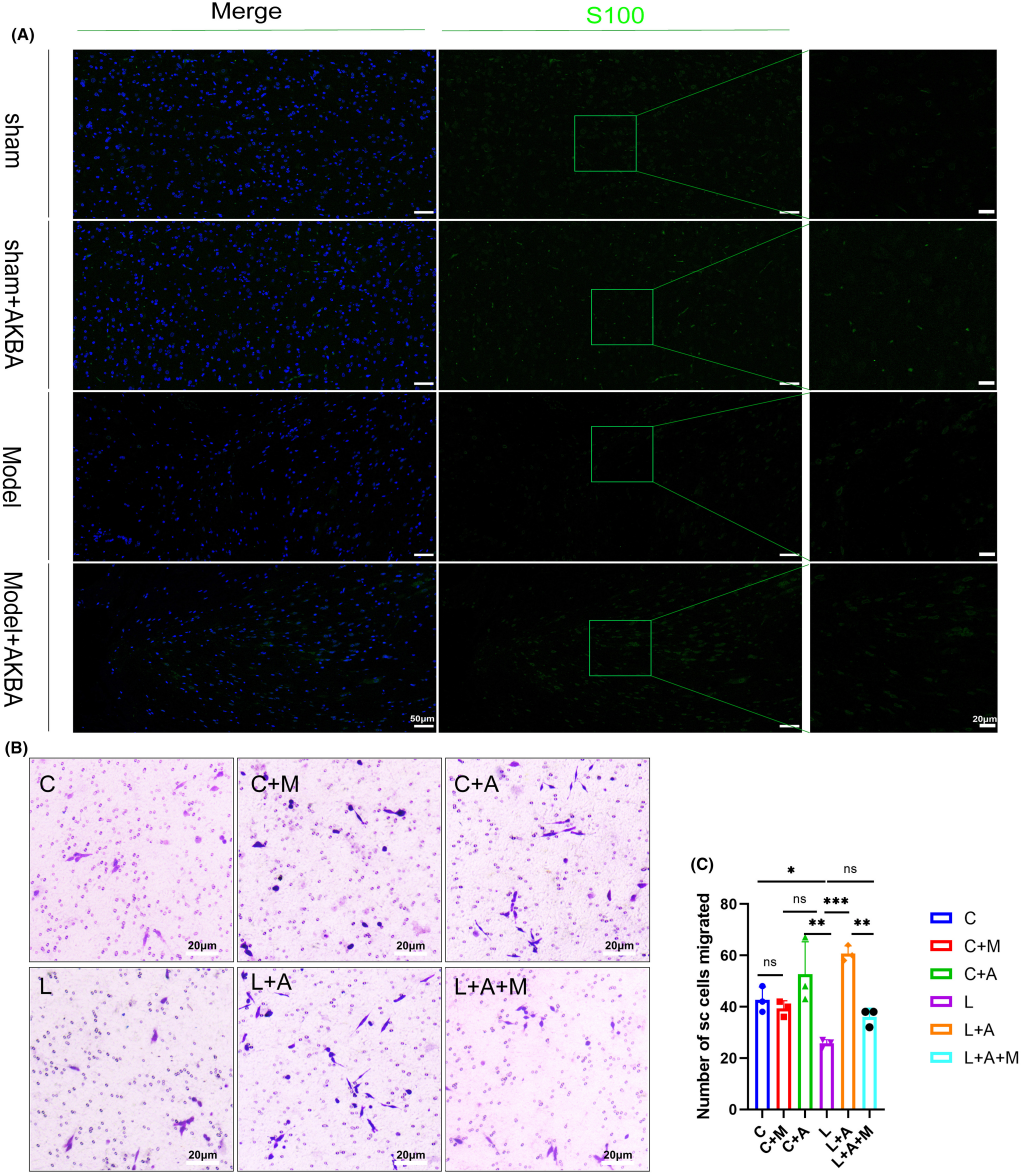
Nrf2 inhibitors attenuate the ability of AKBA to promote Schwann cell migration. (A) Detection of S100 at the site of spinal cord injury. (B) AKBA‐treated macrophage culture promotes the migration ability of Schwann cells. (C) Statistical graph of Schwann cell migration. *p < 0.05, **p < 0.01, ***p < 0.001.

Macrophage transition facilitates the migration of Schwann cells, so, in vivo, we detected the marker protein S‐100 of Schwann cells using the immunofluorescence technique, as shown in Figure 8A. The amount of S‐100 in the model group was less than that in the model + AKBA group, indicating that AKBA can promote the migration of Schwann cells into the injury site. To verify whether AKBA could be causing the migration of Schwann cells by regulating macrophage polarization. We created a co‐culture system of macrophages and Schwann cells using transwell chambers. As shown in Figure 8B,C, we found that the addition of macrophage cultures containing AKBA accelerated the migration of Schwann cells, but the addition of Nrf2 inhibitor weakened the pro‐migration ability of AKBA.

### 3.9 AKBA can promote the polarization of rat macrophage Ram‐bm and affect the migration of Schwann cells

**FIGURE 9:**
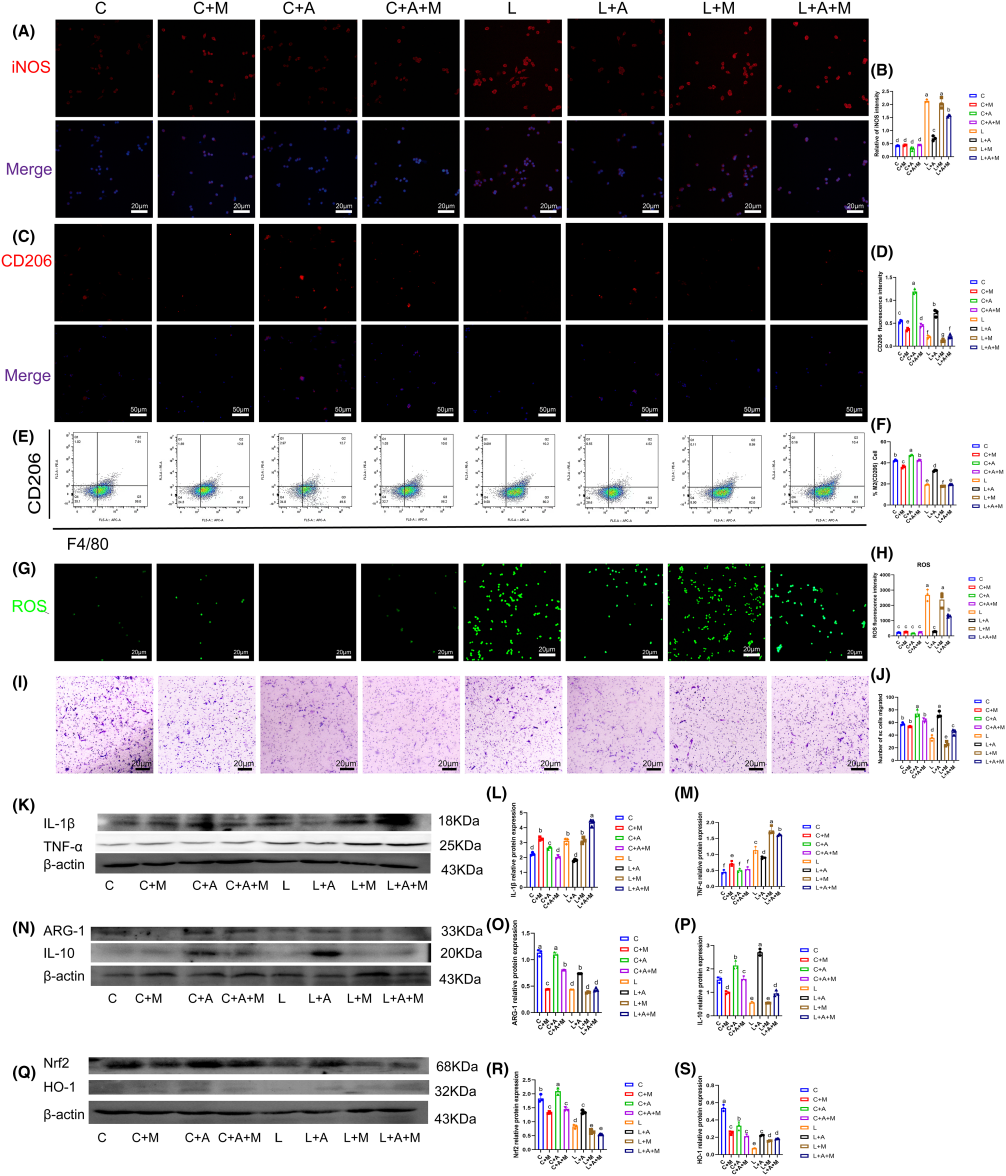
AKBA promotes rat macrophage polarization and Schwann cell migration. (A) iNOS fluorescence image. Red fluorescence indicates iNOS. (B) iNOS fluorescence quantitative analysis chart. (C) CD206 fluorescence image. Red fluorescence indicates CD206. (D) CD206 fluorescence quantitative analysis chart. (E) CD206 flow chart. (F) CD206 flow cytometry analysis diagram. (G) ROS staining picture. (H) ROS fluorescence analysis chart. (I) The migration ability of rat macrophage culture medium on SC cells in each group. (J) Analysis of Schwann cell migration numbers. (K) Protein expression diagram of TNF‐α and IL‐1β. (L) Protein quantitative analysis chart of IL‐1β. (M) Quantitative analysis chart of TNF‐α. (N) Protein expression diagram of ARG‐1 and IL‐10. (O) ARG‐1 protein quantitative analysis chart. (P) Quantitative protein analysis chart of IL‐10. (Q) Protein expression diagram of Nrf2 and HO‐1. Protein quantitative analysis chart of (R) Nrf2. (S) HO‐1 protein quantitative analysis chart. C represents the blank group, C + M represents the blank plus ML385 group, C + A represents the blank plus AKBA group, and C + A + M represents the blank plus AKBA and ML385. L represents the LPS group, L + A represents the LPS + AKBA group, L + M represents the LPS + ML385 group, and L + A + M represents the LPS + AKBA + ML385 group. The lowercase letters above the statistical graph indicate differences. The same letters indicate no significant difference, and different letters indicate significant differences (*p* < 0.05).

It can be seen from Figure 9A,B that compared with group C, C + M, C + A, C + A + M, the fluorescence expression of iNOs in the LPS group increased significantly (*p* < 0.05). Comparable to the LPS group, the L + AKBA group's fluorescence expression of iNOS can be significantly reduced (*p* < 0.05). After adding the Nrf2 inhibitor, the ability of AKBA to reduce iNOS was inhibited. Therefore, this also proves that AKBA reduces the expression level of iNOS through Nrf2. From this, we concluded that AKBA can also reduce the increase in iONS expression levels in rat macrophages caused by LPS. As for whether AKBA can promote the expression level of CD206 in rat macrophages, we can know from Figure 9C,D that AKBA can significantly increase the fluorescence expression of CD206. Compared with group C, the LPS group significantly reduced the expression level of CD206. The addition of AKBA to the LPS group significantly increased the expression level of CD206. After adding the Nrf2 inhibitor, the ability of AKBA to increase the expression of CD206 was inhibited. Therefore, it is shown that AKBA promotes the transformation of rat macrophages through Nrf2. Similarly, we performed flow cytometry analysis on CD206, as shown in Figure 9E,F. We can obtain changes consistent with CD206 fluorescence expression. Compared with the LPS group, the LPS + AKBA group could significantly increase the number of CD206 cells, and this change was inhibited after adding the Nrf2 inhibitor. At the same time, we also explored the changes in ROS. From Figure 9G,H, we can conclude that AKBA can significantly reduce the expression level of ROS. After adding ML385, this ability was inhibited. Therefore, it indicates that AKBA regulates ROS expression level through Nrf2. Similarly, we also verified the expression levels of TNFα and IL‐1β (Figure 9K–M). The results showed that compared with the LPS group, AKBA could significantly reduce the increase in inflammatory factors caused by LPS. After adding ML385, this ability was inhibited. ARG‐1 and IL‐10 were also verified. Consistent with the results in mouse macrophages, AKBA could significantly increase the expression levels of ARG‐1 and IL‐10 (Figure 9N–P). When testing Nrf2 and HO‐1, the results were consistent with the results in mouse macrophages. The AKBA can significantly increase the expression levels of Nrf2 and HO‐1 (Figure 9Q–S). Finally, we also supplemented the migration ability of rat macrophages to SC cells, as shown in Figure 9I,J. The results are the same as those of mouse macrophages, indicating that AKBA promotes the polarization of rat macrophages. The culture medium can promote SC cell migration, but after adding Nrf2 inhibitor, this migration effect is inhibited. Therefore, our results in this part indicate that AKBA can not only promote the polarization of mouse macrophages but also promote the polarization of rat macrophages and promote the migration of Schwann cells.

## 4 DISCUSSION

Repair and reconstruction after spinal cord injury are very difficult, and methylprednisolone is commonly used clinically for treatment, but there are side effects such as kidney damage.^33^ Traditional medicines are a valuable cultural heritage that the ancient people leave to our later generations, to mine the medicinal value of traditional medicines and explore their mechanisms of disease and cure. AKBA, the active ingredient of Boswellia serrata, has anti‐inflammatory and antioxidant effects. After SCI, macrophages and microglia are activated to produce a large amount of ROS, iNOS and other pro‐inflammatory substances, causing oxidative damage and inflammatory reactions in spinal neurons and so on. If the activation of macrophages and microglia can be reduced, it will alleviate oxidative stress and reduce inflammation, thus protecting the spinal cord from inflammatory injury and accelerating injury repair. Our study showed that AKBA was able to protect the spinal cord against secondary injury by reducing the expression of iNOS and the release of ROS, enhancing antioxidant enzyme activity (CAT, GSH‐Px, T‐AOC, SOD), decreasing MDA activity, increasing CD206, Arg‐1, IL‐10 expression, promoting the migration of Schwann cells, alleviating oxidative stress and inflammation.

During the acute response phase of trauma M1 macrophages activate and release high levels of ROS and pro‐inflammatory cytokines (TNF‐α, IL‐1β).^34^ M1 macrophages have an innate immune function to remove foreign microorganisms and wound tissue debris from the injury site and will remain at the injury site thereafter, accelerating the inflammatory response of the spinal cord and impeding the repair of spinal cord injury.^35^ It was shown that gene expression of M1 cells (pro‐inflammatory phenotype) of macrophages was significantly upregulated at the lesion site (3 mm region) and caudally (3 mm) 1 week after SCI.^36^ The results of our study also showed that the expression levels of iNOS, TNF‐α, and IL‐1β in the SCI model group were significantly up‐regulated. Our experimental results found that AKBA can significantly reduce the expression levels of iNOS, TNF‐α, and IL‐1β. HE staining also shows that AKBA can effectively reduce the inflammatory cells at the injury site, which is conducive to the recovery of spinal cord injury. In contrast, M2 macrophages exhibited tissue repair properties and showed diminished production of pro‐inflammatory cytokines.^37^ The phagocytosis of macrophages in the early phase facilitates injury recovery, but after day 3, the accumulation of inflammatory substances such as iNOS and ROS that are continuously secreted by macrophages hinders the repair of injury. Therefore, if we can reduce the activation of M1 macrophages after injury and promote their conversion to M2, we can reduce the secondary damage to the spinal cord caused by inflammation. Our experiments proved that on the 7th day of spinal cord injury, the intervention of AKBA reduced the expression product iNOS of macrophage M1, as well as the corresponding inflammatory factors (TNF‐α, IL‐1β), providing favorable conditions for the injury, And M2 macrophages were also detected, showing that the phenotype of M2 macrophages (CD206, ARG‐1) was significantly increased under the action of AKBA, and the expression of anti‐inflammatory factor IL‐10 was increased. This result is consistent with the experimental results of others that the polarization of macrophages into the M2 type contributes to the recovery of inflammation.^9^, ^38^, ^39^ Thus, it was concluded that AKBA can promote the transformation of macrophages from pro‐inflammatory M1 to anti‐inflammatory M2, which is conducive to reducing inflammation after spinal cord injury, creating a favorable microenvironment for repair after spinal cord injury, and promoting the recovery of spinal cord injury.

Why AKBA promotes the transition of macrophages to M2? Studies have shown that the expression of IL‐10 promotes the transition of macrophages from M1 to M2.^39^ However, the absence of Nrf2/HO‐1 can lead to a decrease in IL‐10 levels in macrophages.^40^ There is also positive feedback between IL‐10 and HO‐1.^41^ On the one hand, IL‐10 induces the production of HO‐1 through STAT‐3 and PI3K pathways;^42^ on the other hand, HO‐1 and CO regulate the production of IL‐10 by activating p38MAPK,^43^ which amplifies the anti‐inflammatory effect of M2 macrophages. Therefore, the overexpression of Nrf2/HO‐1 will promote the expression of IL‐10, thereby promoting the transition of macrophages to M2.^44^ Existing studies have shown that AKBA can significantly promote the expression of Nrf2 and activate HO‐1,^21^, ^45^ but whether it can promote the transformation of macrophages is unknown. Our experimental results answered, not only verifying that AKBA can activate Nrf2/HO‐1, but also further proving that AKBA can increase the expression level of IL‐10 and promote the transformation of macrophages from M1 to M2. After we added the Nrf2 inhibitor ML385, the effect of AKBA on promoting the transformation of macrophages was inhibited, further indicating that AKBA promotes the transformation of macrophages through Nrf2/HO‐1, thereby reducing inflammation and accelerating the repair of spinal cord injuries. This result is consistent with the results that melatonin promotes macrophage transformation through Nrf2/HO‐1.^17^ Compared with M1 macrophages, transformed into M2 macrophages will have less ROS,^46^ and our in vitro results on macrophage ROS have also verified this point. In addition, the activation of Nrf2/HO‐1 can also promote the activity of antioxidant enzymes such as CAT, GSH‐PX, T‐AOC, and SOD, reduce the accumulation of MDA, reduce ROS, help the body resist the influence of oxygen free radicals, reduce the oxidation stress, and promote the repair of damage.

The transformation of macrophages not only reduces inflammation but also promotes the migration of Schwann cells to the damaged spinal cord.^10^ Under normal conditions, Schwann cells do not exist in the spinal cord, however, in rodents^47^ and humans,^48^, ^49^ it was found that Schwann cells migrate to the lesioned area after SCI and secrete neurotrophic factors such as BDNF and NGF, which are very beneficial for the repair of neurons in the spinal cord.^50^ Schwann cells are most likely involved in endogenous repair after spinal cord injury.^13^ Our previous studies have shown that AKBA can promote the expression of BDNF and NGF.^24^ The results of this experiment show that AKBA promotes the migration of Schwann cells through the polarization of macrophages to M2, and secretes neurotrophic factors to promote the repair of spinal cord injuries. Therefore, AKBA not only reduces spinal cord injury by reducing inflammation but also promotes the migration of Schwann cells and facilitates the repair of spinal cord injury.

**FIGURE 10:**
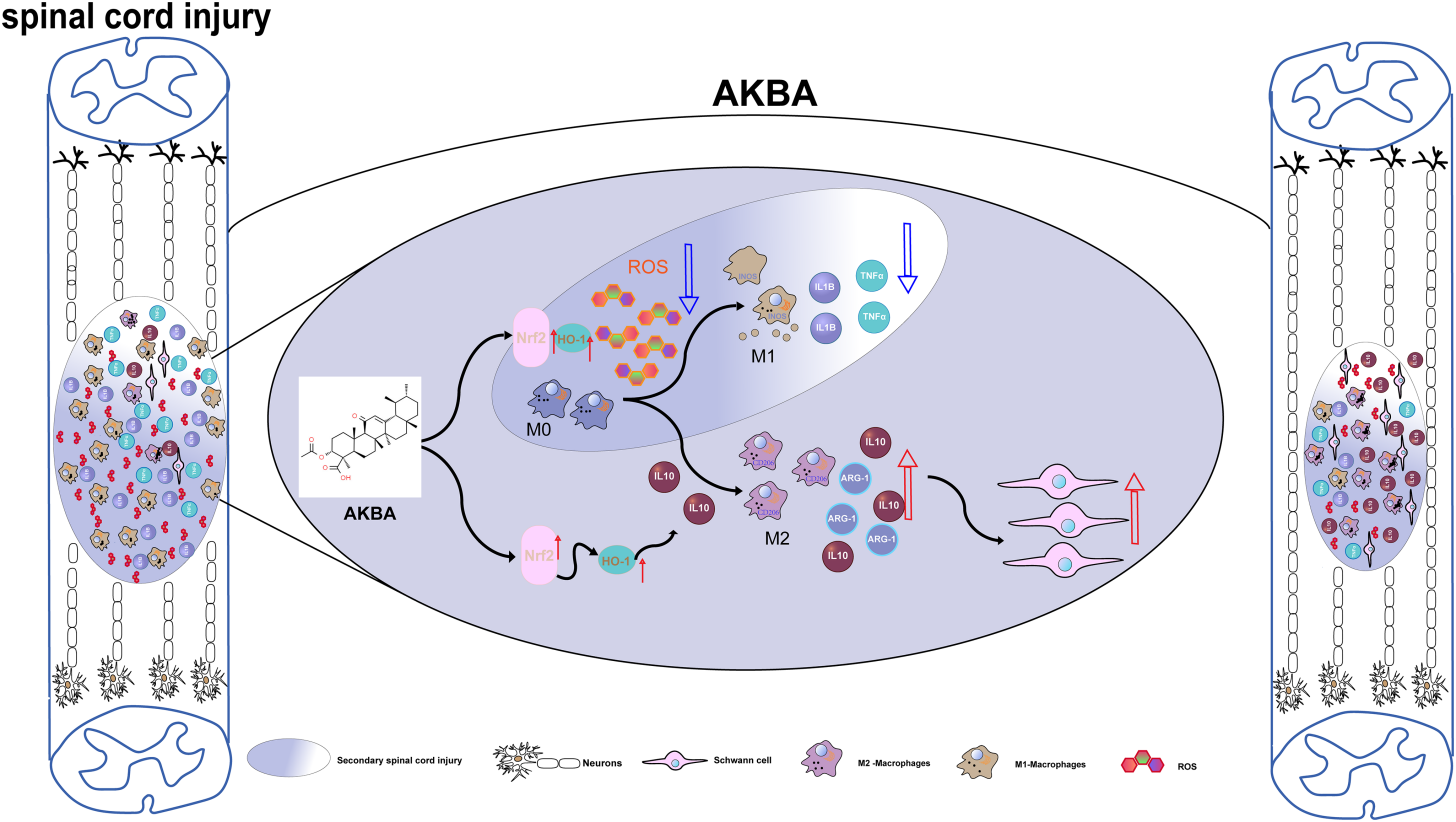
The mechanism of AKBA promoting the spinal cord injury.

In conclusion, AKBA can protect against spinal cord injury by regulating the redox system Nrf2/HO‐1 signaling pathway, reducing ROS generation, and promoting the migration of macrophages to M2 polarization and Schwann cells (Figure 10). In addition, the migration of Schwann cells in the CNS is the key to the formation of CNS neuroma, so we need to further explore the subsequent effects on the spinal cord after the migration of Schwann cells.

## 5 CONCLUSION

AKBA, the most important active ingredient of the natural drug Boswellia serrata, promotes spinal cord injury repair by regulating the Nrf2/HO‐1 signaling pathway, promoting macrophage polarization and Schwann cell migration, creating a microenvironment suitable for injury repair at the spinal cord injury site and accelerating the repair of the damaged spinal cord, and AKBA is expected to become a drug for the treatment of spinal cord injury.

## AUTHOR CONTRIBUTIONS

Yao Wang is the experimental designer and the executive of the experimental research. Zong‐Liang Xiong, Yuncong Qiao, Qi‐Yuan Zhang, Guang‐Hu Zhou, Chong Zhou, Xiang‐lin Ma, and Xiao‐Wen Jiang participated in the experimental research and analysis of the experimental results; Xiao‐Wen Jiang and Wen‐Hui Yu are the creators and leaders of the project, directing the experimental design, data analysis, paper writing, and revision. All authors have read and agreed to the final version of the manuscript.

## FUNDING INFORMATION

This study was supported by the National Natural Science Foundation of China (No. 31972725).

## CONFLICT OF INTEREST STATEMENT

The authors declare no conflicts of interest.

## Data Availability

The data used to support the findings of this study are included in the article.
